# The Antioxidant Status and Concentrations of Coenzyme Q10 and Vitamin E in Metabolic Syndrome

**DOI:** 10.1155/2013/767968

**Published:** 2013-09-03

**Authors:** Chi-Hua Yen, Nae-Cherng Yang, Bor-Jen Lee, Jui-Yuan Lin, Simon Hsia, Ping-Ting Lin

**Affiliations:** ^1^Department of Family and Community Medicine, Chung Shan Medical University Hospital, Taichung 40201, Taiwan; ^2^School of Medicine, Chung Shan Medical University, Taichung 40201, Taiwan; ^3^Center for Education and Research on Geriatrics and Gerontology, Chung Shan Medical University, Taichung 40201, Taiwan; ^4^School of Nutrition, Chung Shan Medical University, Taichung 40201, Taiwan; ^5^Department of Nutrition, Chung Shan Medical University Hospital, Taichung 40201, Taiwan; ^6^The Intensive Care Unit, Taichung Veterans General Hospital, Taichung 40705, Taiwan; ^7^Department of Nutrition and Institute of Biomedical Nutrition, Hungkuang University, Taichung 43346, Taiwan

## Abstract

The purpose of this study was to investigate the levels of coenzyme Q10 and vitamin E and the antioxidant status in subjects with metabolic syndrome (MS). Subjects with MS (*n* = 72) were included according to the criteria for MS. The non-MS group (*n* = 105) was comprised of healthy individuals with normal blood biochemical values. The plasma coenzyme Q10, vitamin E concentrations, lipid profiles, and antioxidant enzymes levels (catalase, superoxide dismutase, and glutathione peroxidase) were measured. The subjects with MS had significantly higher concentrations of plasma coenzyme Q10 and vitamin E than those in the non-MS group, but these differences were not significant after being normalized for triglyceride level. The levels of antioxidant enzymes were significantly lower in the MS group than in the non-MS group. The subjects with the higher antioxidant enzymes activities had significant reductions in the risk of MS (*P* < 0.01) after being adjusted for coenzyme Q10 and vitamin E. In conclusion, the subjects with MS might be under higher oxidative stress resulting in low levels of antioxidant enzyme activities. A higher level of antioxidant enzymes activities was significantly associated with a reduction in the risk of MS independent of the levels of coenzyme Q10 and vitamin E.

## 1. Introduction

Metabolic syndrome (MS) represents a cluster of physiological and anthropometric abnormalities [1] and is recognized as a significant risk factor for cardiovascular disease and type II diabetes [2]. The Third National Health and Nutrition Examination Survey (1988–1994) reported that more than 20% of the adult population in the USA suffered from MS [3, 4]. A recent report from the Elderly Nutrition and Health Survey in Taiwan (NAHSIT) conducted during 1999-2000 noted that 26% of men and 47% of women suffered from MS [5]. The health and dietary transition in Taiwan is beginning to have some similarities with western nations. The NAHSIT (2005–2008) observed that dietary habits in Taiwan were changing, particularly in regard to intake of cakes, sweets, and sugary drinks. The energy intake in young people has increased because of the popularity of fast food chains, and combined with an increasingly sedentary lifestyle, this may have led to the increase in obesity and associated metabolic diseases [6]. The markers of MS, including insulin resistance, type II diabetes, hypertension, dyslipidemia, and visceral obesity, may increase oxidative stress [7–9] and reduce antioxidant defenses [10–12]. Increases in oxidative stress contribute to impaired vascular function, inflammation, thrombosis, and atherosclerosis and ultimately give rise to vascular disease [13].

Coenzyme Q10 and vitamin E are lipid-soluble vitamins known for their excellent antioxidant qualities. The Third National Health and Nutrition Examination Survey indicated that adults with MS have suboptimal concentrations of antioxidants [14]. However, the French SU.VI.MAX (Supplementation en Vitamines et Minéraux AntioXydants) trials followed subjects without MS for 7.5 years and showed that there was no beneficial effect of antioxidant supplementation (such as vitamin E) on the incidence of MS [15]. Coenzyme Q10 (also called ubiquinone) is a lipid-soluble benzoquinone containing 10 isoprenyl units in its side chain. Coenzyme Q10 is a key component of the mitochondrial respiratory chain and is required for adenosine triphosphate (ATP) synthesis [16, 17]. Coenzyme Q10 is an intracellular antioxidant that protects low-density lipoprotein cholesterol (LDL-C) from free radical-induced oxidative damage [18, 19]. It has demonstrated potential cardioprotective properties and reduces the risk of coronary artery disease [20]; however, few studies have examined the concentration of coenzyme Q10 in patients with MS. In the present study, we hypothesize that subjects with MS might be under higher oxidative stress, which influences their antioxidant status. Therefore, the purpose of this study was to investigate the antioxidant status and the levels of coenzyme Q10 and vitamin E in the subjects with MS.

## 2. Materials and Methods

### 2.1. Subjects

This study was a cross-sectional study. We expected the differences in the mean levels of plasma coenzyme Q10 between the MS and non-MS groups to be 0.2 ± 0.3 *μ*mol/L based on the Miles et al. study [21]. The desired power was set at 0.8 to detect a true effect, alpha was set at 0.05 (*α* = 0.05), and a minimum sample size of 50 participants was required for each group. Subjects with MS (*n* = 72) were recruited from the Department of Family and Community Medicine of Chung Shan Medical University Hospital in Taiwan. The inclusion criteria for the MS group were based on the definition published by the Taiwan Bureau of Health Promotion, Department of Health (2007). The subjects were diagnosed with MS if they had 3 of the following 5 characteristics: (1) abdominal obesity (waist circumference ≥90 cm in men and ≥80 cm in women), (2) impaired fasting glucose (≥5.6 mmol/L), (3) hypertriglyceridemia (≥1.7 mmol/L), (4) low high-density lipoprotein cholesterol (HDL-C < 1.0 mmol/L in men and <1.3 mmol/L in women), and (5) increased blood pressure (systolic blood pressure ≥130 mmHg and diastolic blood pressure ≥85 mmHg). The diagnostic criteria for MS of Taiwan are according to the National Cholesterol Education Program Adult Treatment Panel III (ATP III, 2001), International Diabetes Federation (IDF, 2005), and the American Heart Association/National Heart, Lung, and Blood Institute (AHA/NHLBI, 2005) criteria [1, 22, 23]. Therefore, the diagnostic criteria for MS of Taiwan are similar to these international criteria. The subjects who were using antidiabetic, antihypertensive, or lipid-lowering medications were considered to have elevated fasting blood glucose, elevated blood pressures, and dyslipidemia, respectively. The concentration of coenzyme Q10 might be affected by statin therapy [24]; therefore, the subjects with MS who were receiving statin therapy were excluded. The subjects in the non-MS group (*n* = 105) were recruited from the physical examination unit of the hospital. The inclusion criteria for the non-MS group were based on the subjects' medical records from the previous month. The subjects were included in the non-MS group if they did not have a diagnosis of any of the following diseases or conditions: gastrointestinal disorder, hypertension, hyperlipidemia, liver disease, renal disease, diabetes, or other metabolic diseases. The subjects also had to exhibit normal blood biochemical values, including all of the following characteristics: fasting glucose <5.6 mmol/L, blood urea nitrogen <7.9 mmol/L, creatinine <123.8 *μ*mol/L, alkaline phosphate <190 U/L, glutamic oxaloacetic transaminase <35 U/L, and glutamic pyruvate transaminase <45 U/L. The subjects in both MS and non-MS groups who were taking antioxidant vitamin supplements (including coenzyme Q10 and vitamin E supplementation) were excluded from the study. This study was approved by the Institutional Review Board of Chung Shan Medical Hospital in Taiwan, and the written informed consent was obtained from each subject. 

The age, blood pressures, and smoking habits of each subject were recorded. The blood pressure was measured by a sphygmomanometer with repeated measurements for concordance in each subject after resting for at least 5 min. The body weight, height, and waist and hip circumferences of each subject were measured by a trained examiner of the same gender as the study subjects, and the body mass index (kg/m^2^) and the waist to hip ratio were then calculated. The subjects were instructed to complete a 24 h dietary recall of the day before blood sample collection. The nutrient composition was calculated with Nutritionist Professional software (E-Kitchen Business Corp., Taiwan); the nutrient database was based on the Taiwan food composition table (Food and Drug Administration, Department of Health, Taiwan; http://www.doh.gov.tw/FoodAnalysis/ingredients.htm).

### 2.2. Blood Collection and Biochemical Measurement

Fasting venous blood samples (15 mL) were obtained to estimate the levels of fasting glucose, blood lipid profiles, antioxidant enzymes activities, coenzyme Q10, vitamin E, and inflammatory markers. The researchers advised that all subjects fast for 10–12 h before blood collection and avoid exercise for 24 h before blood collection. Blood specimens were collected in vacutainer tubes with and without EDTA as an anticoagulant as necessary. Serum and plasma were prepared and frozen (−80°C) for storage until analysis. Blood lipid parameters (i.e., total cholesterol (TC), triglyceride (TG), LDL-C, and HDL-C) concentrations were measured using an automated biochemical analyzer (Hitachi-7180E, Tokyo, Japan).

Plasma coenzyme Q10 and vitamin E were measured by high-performance liquid chromatography (HPLC) and detected by a UV detector at 275 nm and 292 nm, respectively [25, 26]. Red blood cells (RBCs) were diluted with 25x sodium phosphate buffer for SOD (superoxide dismutase) and GPx (glutathione peroxidase) measurements and 250x sodium phosphate buffer for CAT (catalase) measurement. The methods for measuring CAT, SOD, and GPx in RBCs have previously been described [27–29]; measurements were acquired with a spectrophotometer at 240 nm, 325 nm, and 340 nm, respectively. The RBC protein content was determined based on the Biuret reaction of the BCA kit (Thermo, Rockford, IL, USA). The antioxidant enzymes levels were expressed as units/mg protein. All analyses were performed in duplicate and repeated measurements of the same sample varied by less than 10%. 

### 2.3. Statistical Analyses

The data were analyzed using SigmaPlot software (version 12.0, Systat, San Jose, California, USA). The normal distribution of variables was evaluated using the Shapiro-Wilk test. The differences in demographic and hematological measurement data between the MS and non-MS groups were analyzed using Student's *t*-test or the Mann-Whitney rank sum test. For categorical response variables, differences between the two groups were assessed using the Chi-square test or Fisher's exact test. Adjusted odds ratios with 95% confidence intervals (CI) for MS were calculated from the logistic regression models base on the fifth level (50th percentile) of antioxidant enzymes activities. The data are expressed as the means ± the standard deviations (SD). The results were considered statistically significant at *P* < 0.05. 

## 3. Results

Table 1 shows the demographic data and health characteristics of the subjects. The subjects with MS had significantly higher values of systolic blood pressure, diastolic blood pressure, waist circumference, fasting glucose, TG, LDL-C, and TC/HDL-C and lower HDL-C values than those in the non-MS group. With respect to dietary intake, the subjects with MS had higher fat (*P* < 0.01), saturated fatty acid (*P* = 0.03), and unsaturated fatty acid (*P* = 0.01), and vitamin E (*P* = 0.02) intakes than those in the non-MS group. However, the vitamin E intake was not significantly different between the two groups after normalization by fat intake.

**Figure 1:**
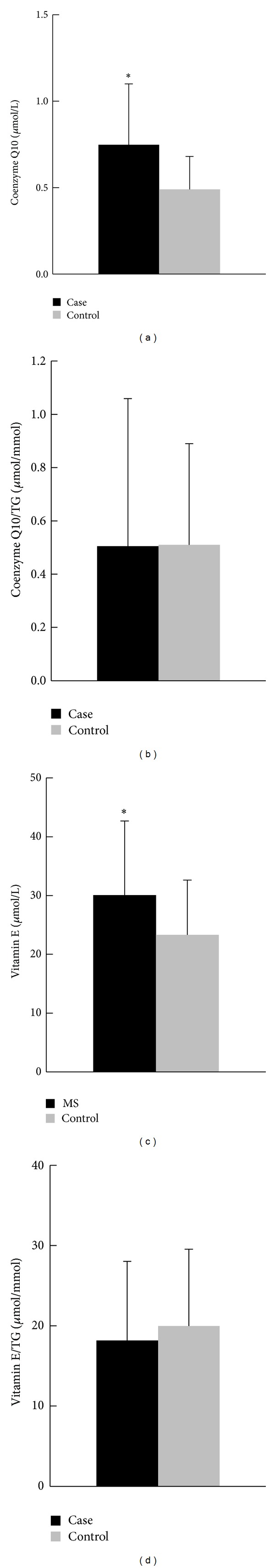
The levels of plasma coenzyme Q10 and vitamin E. *Values were significantly different between case and control groups; *P* < 0.01. TG: triglyceride.

**Figure 2:**
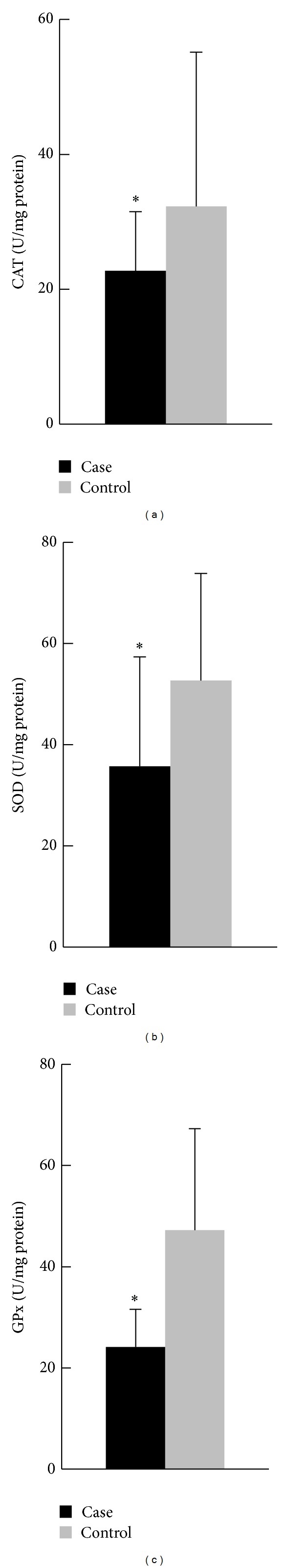
The levels of antioxidant enzymes. *Values were significantly different between case and control groups; *P* < 0.05. CAT: catalase activity; GPx: glutathione peroxidase; SOD: superoxide dismutase.

 The levels of plasma coenzyme Q10, vitamin E, and antioxidant enzymes are shown in Figures 1 and 2. The subjects with MS had significantly higher plasma coenzyme Q10 and vitamin E concentrations (*P* < 0.01). After normalization to TG, the ratio of coenzyme Q10 to TG (*P* = 0.91) and the ratio of vitamin E to TG (*P* = 0.15) were not significantly different between the MS and non-MS groups. With respect to the antioxidant enzymes activities, the subjects with MS had lower CAT (*P* = 0.02), SOD (*P* < 0.01), and GPx activities (*P* < 0.01) than those in the non-MS group. 

 Furthermore, we calculated the odds ratio of MS base on the antioxidant enzymes activities (Table 2). The subjects with higher antioxidant enzymes activities had significant reductions in the risk of MS, even after being adjusted for the potential confounders of MS and the levels of coenzyme Q10 and vitamin E.

## 4. Discussion and Conclusion

The present study showed a statistically significant difference in the antioxidant status and levels of coenzyme Q10 and vitamin E in the subjects with MS. The subjects with MS had significantly higher levels of plasma coenzyme Q10 and vitamin E than those in the non-MS group, but these differences were not statistically significant after normalization to TG. A previous study was conducted by Miles et al. [21], who found that the coenzyme Q10 level was significantly higher in subjects with MS and proposed that an increase in the level of coenzyme Q10 might be a result of the natural antioxidant defense to certain features of MS. The Third National Health and Nutrition Examination Survey [14] also noted that subjects with MS had a significantly higher level of vitamin E compared with non-MS subjects (*P* < 0.01). The concentrations of coenzyme Q10 and vitamin E were closely correlated with lipid profiles. The subjects with MS had a higher level of TG, which may carry the greatest risk of atherogenicity [23], and the levels of antioxidant vitamins (such as coenzyme Q10 and vitamin E) may be increased in triglyceride-rich lipoprotein to provide the lipoprotein lipid with highly efficient antioxidant protection [30]. 

A recent report from NAHSIT (2005–2008) found that the frequency of vegetable and fruit consumption was low in the Taiwanese population [31]. The dietary results showed that the subjects with MS had significantly higher fat, saturated fatty acid, unsaturated fatty acid, and vitamin E intakes, but the vitamin E intake was not significantly different between the two groups after normalization by fat intake (Table 1). Vegetable oil is a major dietary source of vitamin E, and the Taiwanese commonly used vegetable oil (especially soybean oil) in their cooking method (such as stir frying) [6]. In the present study, we did not assess coenzyme Q10 intake, but there was a significant correlation between the level of plasma coenzyme Q10 and fat intake (*r* = 0.17, *P* = 0.04). Meat, fish, nuts, and some oils are the richest nutritional sources of coenzyme Q10 [32]. As a result, a higher fat intake might be an another reason why we observed the higher levels of coenzyme Q10 and vitamin E in the subjects with MS, and the concentrations were not significantly different after adjusted for the lipid. 

Oxidative stress is thought to play an important role in the development of MS [8]. In the present study, we assessed the activities of the major antioxidant enzymes that are directly involved in the neutralization of reactive oxygen species (ROS). The subjects with MS had significantly lower CAT, SOD, and GPx activities (Figure 2), and the subjects with higher antioxidant enzymes activities were significantly associated with a reduction in the risk of MS independent of the levels of coenzyme Q10 and vitamin E (Table 2). Strictly speaking, MS is a type of metabolic disorder rather than a disease. Subjects with MS might be under higher oxidative stress; antioxidant enzymes are the first line of defense against ROS and may decrease to adjust for higher levels of oxidative stress [12]. In addition, MS subjects in general were typically abdominally obese. Obesity is also an oxidative burden that may lead to the reduction of antioxidant enzymes activities [33].

There are some limitations of the present study. First, this study was cross-sectional; therefore, no causal relationship could be defined. Larger prospective studies are required to establish the relationship between antioxidant status in subjects with MS. Second, we did not assess coenzyme Q10 intake because of insufficient data in the nutrients databank. Further developments in analytical chemistry (HPLC) are needed to provide the better information about the coenzyme Q10 content in various foods. Third, we preformed the 24 h dietary recall to evaluate the nutrients intake in this study; however, the MS does not derive from a short-term shock. Further study is needed to clarify the differences of usual dietary intake between MS and non-MS subjects by long-term dietary assessment.

In conclusion, the subjects suffering from MS might be under higher oxidative stress, resulting in low levels of antioxidant enzyme activities. A higher level of antioxidant enzymes activities was significantly associated with a reduction in the risk of MS independent of the levels of coenzyme Q10 and vitamin E. 

## Figures and Tables

**Table 1:** Characteristics of subjects.

	MS (*n* = 72)	Non-MS (*n* = 105)
Male/female (*n*)	43/29	52/53
Age (y)	53.3 ± 11.6^1^	52.0 ± 8.1
Systolic blood pressure (mmHg)	141.9 ± 11.8*	118.8 ± 16.8
Diastolic blood pressure (mmHg)	88.5 ± 10.3*	77.7 ± 9.8
Waist circumference (cm)	96.0 ± 12.4*	80.6 ± 13.6
Fasting glucose (mmol/L)	7.5 ± 2.6*	5.2 ± 1.1
TC (mmol/L)	4.9 ± 1.0	5.1 ± 0.9
TG (mmol/L)	1.9 ± 0.9*	1.3 ± 0.6
LDL-C (mmol/L)	3.2 ± 0.9*	2.9 ± 0.7
HDL-C (mmol/L)	1.2 ± 0.3*	1.4 ± 0.4
TC/HDL-C	4.4 ± 1.2*	3.9 ± 1.2
*Dietary intake *		
Energy (Kcal/d)	2002.8 ± 529.9	1905.0 ± 694.4
Protein (g/d)	67.7 ± 20.8	76.3 ± 36.9
Percentage of total calories	(13.6%)	(16.0%)
Fat (g/d)	74.7 ± 27.3*	60.5 ± 32.3
Percentage of total calories	(33.4%)	(28.6%)
Carbohydrate (g/d)	264.1 ± 76.3	265.8 ± 104.1
Percentage of total calories	(53.0%)	(55.8%)
Polyunsaturated fatty acid (g/d)	26.9 ± 13.2*	21.2 ± 12.3
Percentage of total calories	(12.1%)	(10.0%)
Monounsaturated fatty acid (g/d)	22.6 ± 11.0*	18.8 ± 12.6
Percentage of total calories	(10.2%)	(8.9%)
Saturated fatty acid (g/d)	20.4 ± 10.3*	17.2 ± 11.5
Percentage of total calories	(9.2%)	(8.1%)
Vitamin E (mg *α*-T.E./d)	6.6 ± 3.4*	5.5 ± 3.4
Vitamin E/fat (mg *α*-T.E./g)	0.1 ± 0.0	0.1 ± 0.1

^1^Mean ± SD. *Values were significantly different between case and control groups.

HDL-C: high-density lipoprotein-cholesterol; LDL-C: low density lipoprotein-cholesterol; TC: total cholesterol; TG: triglyceride.

**Table 2:** The odds ratios of metabolic syndrome based on the antioxidant enzymes activities.

	Odds ratio (95% CI)	*P* value
CAT < 22.5 U/mg protein	1.00	—
CAT ≥ 22.5 U/mg protein		
Model 1^1^	0.45 (0.24–0.84)	0.01
Model 2^2^	0.29 (0.11–0.75)	0.01
SOD < 42.2 U/mg protein	1.00	—
SOD ≥ 42.2 U/mg protein		
Model 1	0.25 (0.13–0.47)	<0.01
Model 2	0.29 (0.11–0.75)	0.01
GPx < 34.1 U/mg protein	1.00	—
GPx ≥ 34.1 U/mg protein		
Model 1	0.03 (0.01–0.08)	<0.01
Model 2	0.06 (0.02–0.19)	<0.01

^1^None adjusted.

^2^Adjusted for age, gender, waist circumferences, triglyceride, coenzyme Q10, and vitamin E.

CAT: catalase activity; CI: confidence interval; GPx: glutathione peroxidase; SOD: superoxide dismutase.

## References

[1] Cleeman JI (2001). Executive summary of the third report of the National Cholesterol Education Program (NCEP) expert panel on detection, evaluation, and treatment of high blood cholesterol in adults (adult treatment panel III). *Journal of the American Medical Association*.

[2] Ferrannini E, Haffner SM, Mitchell BD, Stern MP (1991). Hyperinsulinaemia: the key feature of cardiovascular and metabolic syndrome. *Diabetologia*.

[3] Ford ES, Giles WH (2003). A comparison of the prevalence of the metabolic syndrome using two proposed definitions. *Diabetes Care*.

[4] Park Y-W, Zhu S, Palaniappan L, Heshka S, Carnethon MR, Heymsfield SB (2003). The metabolic syndrome: prevalence and associated risk factor findings in the US population from the Third National Health and Nutrition Examination Survey, 1988–1994. *Archives of Internal Medicine*.

[5] Lee LT, Chang YC, Huang KC, Pan WH, Chen CY (2005). The prevalence survey of metabolic syndrome of the elderly in Taiwan. *Taiwan Geriatrics & Gerontology*.

[6] Pan W-H, Wu H-J, Yeh C-J (2011). Diet and health trends in Taiwan: comparison of two nutrition and health surveys from 1993–1996 and 2005–2008. *Asia Pacific Journal of Clinical Nutrition*.

[7] Ceriello A, Quatraro A, Giugliano D (1993). Diabetes mellitus and hypertension: the possible role of hyperglycaemia through oxidative stress. *Diabetologia*.

[8] Giugliano D, Ceriello A, Paolisso G (1995). Diabetes mellitus, hypertension, and cardiovascular disease: which role for oxidative stress?. *Metabolism*.

[9] West IC (2000). Radicals and oxidative stress in diabetes. *Diabetic Medicine*.

[10] Antoniades C, Tousoulis D, Tentolouris C, Toutouzas P, Stefanadis C (2003). Oxidative stress, antioxidant vitamins, and atherosclerosis. From basic research to clinical practice. *Herz*.

[11] Stocker R, Keaney JF (2004). Role of oxidative modifications in atherosclerosis. *Physiological Reviews*.

[12] Penckofer S, Schwertz D, Florczak K (2002). Oxidative stress and cardiovascular disease in type 2 diabetes: the role of antioxidants and pro-oxidants. *The Journal of Cardiovascular Nursing*.

[13] Giugliano D (2000). Dietary antioxidants for cardiovascular prevention. *Nutrition, Metabolism and Cardiovascular Diseases*.

[14] Ford ES, Mokdad AH, Giles WH, Brown DW (2003). The metabolic syndrome and antioxidant concentrations: findings from the Third National Health and Nutrition Examination Survey. *Diabetes*.

[15] Czernichow S, Vergnaud A-C, Galan P (2009). Effects of long-term antioxidant supplementation and association of serum antioxidant concentrations with risk of metabolic syndrome in adults. *American Journal of Clinical Nutrition*.

[16] Ernster L, Dallner G (1995). Biochemical, physiological and medical aspects of ubiquinone function. *Biochimica et Biophysica Acta*.

[17] Bhagavan HN, Chopra RK (2006). Coenzyme Q10: absorption, tissue uptake, metabolism and pharmacokinetics. *Free Radical Research*.

[18] Alleva R, Tomasetti M, Battino M, Curatola G, Littarru GP, Folkers K (1995). The roles of coenzyme Q10 and vitamin E on the peroxidation of human low density lipoprotein subfractions. *Proceedings of the National Academy of Sciences of the United States of America*.

[19] Singh U, Devaraj S, Jialal I (2007). Coenzyme Q10 supplementation and heart failure. *Nutrition Reviews*.

[20] Lee BJ, Lin YC, Huang YC, Ko YW, Hsia S, Lin PT (2012). The relationship between coenzyme Q10, oxidative stress, and antioxidant enzymes activities and coronary artery disease. *The Scientific Word Journal*.

[21] Miles MV, Horn PS, Morrison JA, Tang PH, DeGrauw T, Pesce AJ (2003). Plasma coenzyme Q10 reference intervals, but not redox status, are affected by gender and race in self-reported healthy adults. *Clinica Chimica Acta*.

[22] Alberti KGMM, Zimmet P (2005). The metabolic syndrome—a new worldwide definition. *The Lancet*.

[23] Grundy SM, Cleeman JI, Daniels SR (2005). Diagnosis and management of the metabolic syndrome: an American Heart Association/National Heart, Lung, and Blood Institute scientific statement. *Circulation*.

[24] Mabuchi H, Higashikata T, Kawashiri M (2005). Reduction of serum ubiquinol-10 and ubiquinone-10 levels by atorvastatin in hypercholesterolemic patients. *Journal of Atherosclerosis and Thrombosis*.

[25] Littarru GP, Mosca F, Fattorini D, Bompadre S Method to assay coenzyme Q10 in blood plasma or blood serum.

[26] Hatam LJ, Kayden HJ (1979). A high-performance liquid chromatographic method for the determination of tocopherol in plasma and cellular elements of the blood. *Journal of Lipid Research*.

[27] Paglia DE, Valentine WN (1967). Studies on the quantitative and qualitative characterization of erythrocyte glutathione peroxidase. *The Journal of Laboratory and Clinical Medicine*.

[28] Marklund S, Marklund G (1974). Involvement of the superoxide anion radical in the autoxidation of pyrogallol and a convenient assay for superoxide dismutase. *European Journal of Biochemistry*.

[29] Aebi H (1984). Catalase in vitro. *Methods in Enzymology*.

[30] Mohr D, Stocker R (1994). Radical-mediated oxidation of isolated human very-low-density lipoprotein. *Arteriosclerosis and Thrombosis*.

[31] Chen K-J, Pan W-H, Lin Y-C, Lin B-F (2011). Trends in folate status in the Taiwanese population aged 19 years and older from the Nutrition and Health Survey in Taiwan 1993–1996 to 2005–2008. *Asia Pacific Journal of Clinical Nutrition*.

[32] Pravst I, Žmitek K, Žmitek J (2010). Coenzyme Q10 contents in foods and fortification strategies. *Critical Reviews in Food Science and Nutrition*.

[33] Karaouzene N, Merzouk H, Aribi M (2011). Effects of the association of aging and obesity on lipids, lipoproteins and oxidative stress biomarkers: a comparison of older with young men. *Nutrition, Metabolism and Cardiovascular Diseases*.

